# Prevalence of Digital Eye Strain During the COVID-19 Pandemic Among Adolescent Schoolchildren in Chengalpattu District

**DOI:** 10.7759/cureus.98998

**Published:** 2025-12-11

**Authors:** Kavya Palanisamy, Raja D, Rajeswari N, Sri Varsha, Rajan Edward Daniel Thomas

**Affiliations:** 1 Community Medicine, Karpagam Faculty of Medical Sciences and Research, Coimbatore, IND; 2 Community Medicine, Chettinad Hospital and Research Institute, Chettinad Academy of Research and Education, Chengalpattu, IND; 3 Community Medicine, Sree Balaji Medical College and Hospital, Chennai, IND

**Keywords:** adolescent school children, digital devices, digital eye strain, online classes, screen time

## Abstract

Introduction

Children were exposed to excessive screen time during the COVID-19 pandemic due to online classes, which led to increased use of digital devices for social connections and recreation. Children who continue to use digital devices for longer periods at younger ages are at higher risk of developing myopia and digital eye strain (DES), which remains a public health concern even after schools have reopened.

Aims

To determine the prevalence of DES and its associated factors among adolescent schoolchildren (11-17 years) in the Chengalpattu District.

Settings and design

A cross-sectional study was carried out in schools in Chengalpattu District.

Materials and methods

To assess the prevalence of DES and its contributing factors, a semi-structured questionnaire was used. The study included all students in schools selected using simple random sampling.

Statistical analysis

Data were analyzed using IBM SPSS Statistics version 21. For categorical variables, Fisher’s exact test and the chi-square test were used, and p < 0.05 was considered statistically significant.

Results

The mean age of the 546 participants was 12.77 ± 1.7 years. During the lockdown, the average time spent using digital devices was 6.7 ± 2.5 hours. Overall, 93% (n = 508) of children had screen time >5 hours during the lockdown. About 51.5% of adolescents had DES.

Conclusions

This study underscores the need to reduce DES among children through eye health education and by promoting periodic eye check-ups.

## Introduction

Extended school closures brought on by COVID-19 forced educational institutions to implement virtual learning for students, which increased the time children spent using screens for social and recreational purposes [1,2]. Excessive screen time has been implicated in many negative health outcomes [3,4]. The blue light emitted by digital devices can lead to digital eye strain (DES) [5,6]. It is evident that DES among children is a crucial yet neglected public health concern. Therefore, the purpose of this study was to ascertain the prevalence of DES and its associated factors among schoolchildren in Chengalpattu.

## Materials and methods

Study setting and participants

A cross-sectional study was carried out in the schools of Chengalpattu District from July to October 2023. All adolescents aged 11-17 years who provided their assent and whose parents provided consent comprised the study population. Children with infectious eye diseases, inflammatory eye diseases, or anatomical ocular malformations were excluded.

Sampling technique

As per Mohan A et al. [7], using a prevalence of DES among adolescent schoolchildren of 50%, with an absolute precision of 5% and a 95% CI, the required sample size was 384. Accounting for a 10% non-response rate, the minimum sample size was 422. From the list of higher secondary schools (6th to 12th standard), two schools were selected by simple random sampling. After obtaining administrative approval from the selected schools, all eligible students present at the time of the study who fulfilled the inclusion criteria were included. The outcome of this study was to determine the prevalence of DES during the COVID-19 era and the risk factors linked to it among adolescent schoolchildren in Chengalpattu District.

Data collection methods and tools

Before commencing the study, Institutional Human Ethics Committee approval was obtained (Ref. no: IHEC-I/1115/22). Informed consent was collected from parents, and assent was obtained from participants after they were informed about the research in the local language. Basic information and sociodemographic details were gathered using a pretested semi-structured questionnaire. The Computer Vision Syndrome Questionnaire (CVS-Q), a validated tool, was used to evaluate DES.

Data entry and statistical analyses

Data were gathered using a semi-structured questionnaire and entered into a Microsoft Excel spreadsheet, then analyzed using IBM SPSS v21. Mean and SD were used to describe quantitative variables, while frequency and percentage described qualitative variables. Statistical significance was assessed using the chi-square test and Fisher’s exact test. A p-value < 0.05 was considered statistically significant.

Operational definitions

Adolescents

Adolescence is the phase of life between childhood and adulthood, from ages 10 to 19 [8]. They are categorized into:

Younger adolescents: The lives of younger adolescents, defined here to encompass girls and boys from 10 to 14 years of age, are characterized by profound biological, cognitive, emotional, and social changes associated with the passage through puberty [9].

Older adolescents: They encompass girls and boys from 15 to 19 years of age [10].

Computer Vision Syndrome Questionnaire

The CVS-Q is a validated tool developed by Seguí M et al. [11] to estimate ocular symptoms related to digital device exposure. The total score is obtained using the formula:

$&quot;Score\sum_{i=1}^{16}\text{}(frequency\text{ of symptom occurrence})i\times \text{(intensity of symptom)}i&quot;$ 

The results for each symptom were recorded using frequency × intensity as 0 = 0, 1-2 = 1, and 4 = 2. The sum of the symptom scores provides the DES score; a score ≥ 6 indicates that the child has DES. DES severity was classified as mild (DES score: 6-12), moderate (13-18), and severe (19-32) [11].

## Results

During the lockdown, all research participants took online classes for academic credit. In addition, approximately 5.8% of participants were enrolled in other online courses, with 4.9% taking them for academic purposes (e.g., math and English lessons) and 0.9% for non-academic purposes (e.g., yoga and music classes) (Table 1).

**Table 1:** Socio-demographic and online classes details of study participants (N=546).

Characteristics	N	%
Mean age ± SD	12.77 ± 1.7 years	
Age group		
11-14 years	456	83.5
15-17 years	90	16.5
Gender		
Male	292	53.5
Female	254	46.5
Class		
6th-8th	358	65.5
9th-10th	120	22
11th-12th	68	12.5
Socioeconomic status (Modified BG Prasad classification)		
Upper class	445	81.5
Upper middle class	91	16.7
Lower middle class	9	1.6
Upper lower class	1	0.2
Details of online classes		
Children who attended online classes	546	100
Children currently attending online classes	32	5.8
Breaks between classes		
Present	518	94.9
Absent	28	5.1
Number of breaks between classes		
No breaks	28	5.1
1-2 breaks	386	70.7
>2 breaks	132	24.2

During lockdown, about 93% (N = 508) of children spent more than five hours in front of a screen. Screen time of ≥5 hours per day was highest during the lockdown (93%) and was still reported by half of the participants after the lockdown (50.4%). Most participants used smartphones (74.2%) for online classes (Table 2).

**Table 2:** Details of digital device use among study participants (N = 546).

Variables	N	%
Duration of digital device usage (mean ± SD)		
Before lockdown	1.37 ± 0.6 hours	
During lockdown	6.78 ± 2.5 hours	
After lockdown	3.01 ± 1.8 hours	
Distance of digital device from eyes during online classes		
<1.5 feet	245	44.9
≥1.5 feet	301	55.1
Screen time per day		
Before lockdown (mean ± SD)	4.5 ± 1.9 hours	
<5 hours	303	55.5
≥5 hours	243	44.5
During lockdown (mean ± SD)	8.9 ± 0.9 hours	
<5 hours	38	7
≥5 hours	508	93
After lockdown (mean ± SD)	4.8 ± 2.3 hours	
<5 hours	271	49.6
≥5 hours	275	50.4
Digital devices used for online classes		
Smartphone	405	74.2
Desktop	16	2.9
Laptop	70	12.8
Tablet/iPad	55	10.2

Playing video games (55.1%), watching television (67.4%), and other activities on smartphones (58.6%) increased during lockdown compared with before and after lockdown. Outdoor playtime was drastically reduced to less than 1 hour among 95.1% of children during lockdown. Recreational activities such as drawing and writing (86.3%) increased during and after lockdown. Reading time of more than 4 hours (88.6%) among children also increased during lockdown. The most common symptoms were headache (59.7%, N = 326) and itching (56.1%, N = 306). Worsening of eyesight (16.8%, N = 93) was the least common presenting symptom. Symptoms most often reported as “always” with severe intensity were eye pain (2.2%) and dryness (2.0%) (Table 3).

**Table 3:** Frequency and intensity of digital eye strain (DES) symptoms (N = 546). DES: Digital eye strain.

Symptoms of digital eye strain	Frequency of DES symptoms (%)			Intensity of DES symptoms (%)	
	Never	Occasional - moderate	Occasional - severe	Always - moderate	Always - severe
Burning	50	39.2	8.4	1.8	0.5
Itching	44	45.6	5.9	3.5	1.1
Foreign body sensation	67	23.3	5.5	3.8	0.4
Watering/tearing	47.3	39.2	11	1.3	1.3
Excessive blinking	71.8	21.1	5.9	0.9	0.4
Redness	52.7	38.3	6.8	1.8	0.4
Eye pain	51.5	38.1	6.4	1.8	2.2
Heaviness in eyelids	76	19.8	2.9	0.9	0.4
Dryness	80	14.5	2.7	0.7	2
Blurring of vision	74.9	17.9	3.5	2.4	1.3
Double vision	78.6	18.3	1.8	1.3	0
Increased sensitivity to light	58.4	28.6	9.2	2	1.8
Headache	40.3	42.3	8.1	7.7	1.6
Worsening of eyesight	83	11.2	3.8	1.8	0.2

**Figure 1:**
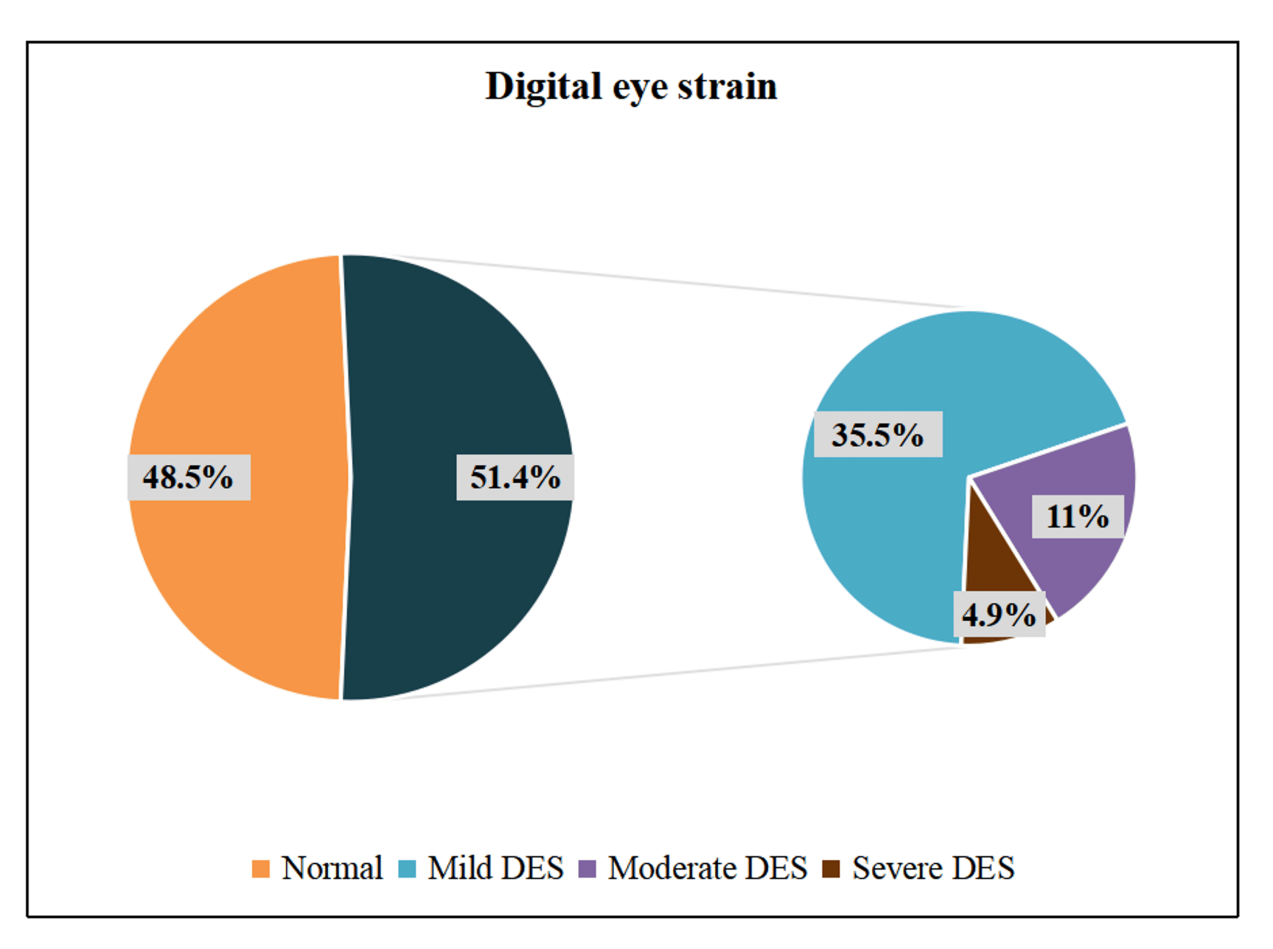
Frequency distribution of DES severity among adolescent schoolchildren (N = 546). DES: Digital eye strain.

Figure 1 shows that more than half of the participants had DES (51.5%), of whom 35.5% had mild DES, 11% had moderate DES, and 4.9% had severe DES. Older adolescents (59%) and females (56.6%) had more DES.

Factors significantly associated with DES included: students of parents who had school education; a positive family history of wearing glasses (p = 0.001); being in 11th and 12th standards (p = 0.018); currently attending online classes (p = 0.001); having no breaks between online classes (p = 0.041); difficulty reading books (p = 0.001) and reading the board (p = 0.001); smartphone use (p = 0.031); and using a device at <1.5 feet distance (51.2%, p = 0.002). About 55.6% (N = 205) of participants who used smartphones had DES. Participants who used screens for five or more hours a day were significantly more likely to develop DES both before and during lockdown (p = 0.010 and p = 0.030, respectively).

More than half of the participants who watched television for ≥2 hours before lockdown had DES (N = 180, 64.1%). During lockdown, the highest numbers with DES were among those who played video games for ≥2 hours (N = 171, 60.9%) and those who had online classes for ≥2 hours (N = 259, 92.2%). After the lockdown, attending online classes for ≥2 hours was associated with DES (N = 13, 92.9%). Analyzing screen-time types showed significant associations with DES for watching television before lockdown (p = 0.001); online classes (p = 0.0001) and video games (p = 0.006) during lockdown; and attending online classes after lockdown (p = 0.0001) (Table 4).

**Table 4:** Factors associated with digital eye strain among study participants (N = 546). *p < 0.05 considered statistically significant;^ a^ Fisher’s exact test applied. DES: Digital eye strain.

Variables	Normal n (%)	DES n (%)	Chi-square value	p-value
School classes				
6th-8th	182 (50.8)	176 (49.2)	32.715	0.018*
9th-10th	58 (48.3)	62 (51.7)		
11th-12th	25 (36.7)	43 (63.3)		
Children attending online classes now				
Yes	5 (15.6)	27 (84.4)	23.863	0.0001*
No	260 (50.5)	254 (49.5)		
Digital devices				
Smartphone	180 (44.4)	225 (55.6)	18.338	0.031*
Desktop	11 (68.8)	5 (31.2)		
Laptop	43 (61.4)	27 (38.6)		
Tablet/iPad	32 (58.2)	23 (41.8)		
Distance of digital device use				
< 1.5 feet	101 (41.2)	144 (58.8)	21.309	0.002*
≥ 1.5 feet	164 (54.4)	137 (45.6)		
Screen time per day - before lockdown				
Watching TV < 2 hours	134 (50.5)	101 (35.9)	11.896	0.001*
≥ 2 hours	131 (49.5)	180 (64.1)		
During lockdown				
Online class < 2 hours	22 (8.3)	22 (7.8)	21.984	0.0001*
≥ 2 hours	243 (91.7)	259 (92.2)		
Video games (during lockdown)				
< 2 hours	135 (50.9)	110 (39.1)	7.673	0.006*
≥ 2 hours	130 (49.1)	171 (60.9)		
After lockdown				
Online class < 2 hours	264 (49.4)	270 (50.6)	13.550ᵃ	0.0001*
≥ 2 hours	1 (7.1)	13 (92.9)		

## Discussion

About 59% of participants in this study had a positive family history of wearing spectacles, higher than the 27% reported by Vishnuprasad R et al. [12]. The difference may be because that study included only parents and siblings, whereas ours also included grandparents along with parents and siblings. According to Mohan A et al. [7], the mean age at which children started wearing glasses was 9.39 ± 3.3 years, which was similar to our study, where the mean age at which children started wearing glasses was 10.28 ± 1.45 years [7]. Our study found that 100% of participants had taken online classes during lockdown, which accords with Mohan A et al., who reported 96.3% [7]. In this study, after lockdown, 5.8% of participants were still attending online classes, of which 4.9% were for academic and 0.9% for non-academic reasons. To our knowledge, no other studies report the proportion of children currently attending online classes; the present study assessed this determinant.

In line with Moitra P et al. (64.9%) and Moon JH et al. (61.3%), 74.2% of participants in this study reported using a smartphone [13,14]. The average duration of digital device use in our study was 1.37 ± 0.6 hours before the lockdown and 6.78 ± 2.5 hours during the lockdown, which is broadly consistent with Mohan A et al., who reported 1.9 ± 1.1 hours before and 3.9 ± 1.9 hours during lockdown [7]. Mohan A et al. and Ichhpujani P et al. found that 31.6% and 44% of participants, respectively, used devices at a distance of less than 18 inches (1.5 feet) from the eyes; in our study, 44.9% reported a distance of < 1.5 feet [7,15]. Screen time of >5 hours per day before lockdown and during lockdown was 1.8% and 36.9%, respectively, in previous studies, which is higher in our study at 44.5% (before) and 93% (during). This difference may be because previous studies assessed screen time based on a single device, whereas our study accounted for multiple device use.

Among the digital devices used for online classes in this study, 74.2% of the children used smartphones. This was higher than in the Moitra P et al. study, where 68.5% of children used smartphones, and the Mohan A et al. study, where 61.7% used smartphones [7,14]. The difference may be because the previous studies included multiple device use during online classes, whereas this study captured single (primary) device use.

In this study, 51.5% of participants had DES, which is consistent with the findings of Mohan A et al. (50.2%) and Courtin R et al. (54%), while other studies reported 17.9% and 19.7% [7,16]. The higher prevalence might be due to prolonged digital device use during the COVID-19 pandemic with the introduction of virtual learning. The most prevalent DES symptoms reported by more than half of participants were headache and itching, in line with Shantakumari N et al. (53.3%), Babu JV et al. (53%), and Mohan A et al. (53.9%) [7,17,18]. Older adolescents in our study had more DES than younger adolescents, similar to the findings of Moon JH et al. and Mohan A et al. [7,13]. An upsurge in visual demand due to increased near work in older adolescents may explain this. Consistent with Babu JV et al. and Shima M et al., girls experienced more DES symptoms than boys [17,19].

A strength of this study is that it assessed DES among children and compared various factors before, during, and after lockdown. Children identified with DES were referred to a nearby higher eye center for further ophthalmic examination. Limitations include potential recall bias due to self-reporting; to mitigate this, data collection and ocular examinations were conducted within one year of school reopening after lockdown. Additionally, the lack of further regression analysis limits our ability to rule out confounding factors.

## Conclusions

More than half of the participants had DES, associated with online classes, increased use of digital devices, and close viewing distances during the pandemic. These findings underscore the need to reduce visual demands among children through eye-health education and to promote periodic eye check-ups, which aid early diagnosis and treatment, given the limited eye-health knowledge among children, parents, and teachers. Further research on strategies to reduce DES should be encouraged.

## Appendices

Appendix 1

**Table 5:** Digital eye strain (DES) questionnaire.

Family details	Education		Occupation	Income
Father/Guardian				
Mother/Guardian				
Total family income (monthly)				
Total number of family members				
Per-capita income				

Online class (please tick the appropriate box)	Attended in the Past		Attending now	
	Yes	No	Yes	No
If yes, mention the reason				

Indicate duration (hours per day)	Before lockdown				During lockdown				After lockdown			
	<1 hour	1-2 hour	2-4 hour	>4 hours	<1 hour	1-2 hour	≥2 hour	>4 hours	<1 hour	1-2 hour	≥2 hour	>4 hours
Outdoor playing												
Online classes												
Video game												
Watching television												
School hours												
Other activity on smartphone												
Reading time												
Drawing and writing time												
